# Spatial distribution of conspecific genotypes within chimeras of the branching coral *Stylophora pistillata*

**DOI:** 10.1038/s41598-021-00981-5

**Published:** 2021-11-19

**Authors:** Gabriele Guerrini, Dor Shefy, Jacob Douek, Nadav Shashar, Tamar L. Goulet, Baruch Rinkevich

**Affiliations:** 1 Israel Oceanography and Limnological Research, National Institute, of Oceanography, Tel-Shikmona, P.O. Box 9753, 3109701 Haifa, Israel; 2 Department of Life Sciences, Eilat Campus, Ben Gurion University of the Negev, Eilat, Israel; 3 The Interuniversity Institute for Marine Science, 88000 Eilat, Israel; 4 Department of Biology, University of Mississippi, P.O. Box 1848, University, MS 38677-1848 USA

**Keywords:** Ecology, Evolution

## Abstract

Chimerism is a coalescence of conspecific genotypes. Although common in nature, fundamental knowledge, such as the spatial distribution of the genotypes within chimeras, is lacking. Hence, we investigated the spatial distribution of conspecific genotypes within the brooding coral *Stylophora pistillata*, a common species throughout the Indo-Pacific and Red Sea. From eight gravid colonies, we collected planula larvae that settled in aggregates, forming 2–3 partner chimeras. Coral chimeras grew in situ for up to 25 months. Nine chimeras (8 kin, 1 non-related genotypes) were sectioned into 7–17 fragments (6–26 polyps/fragment), and genotyped using eight microsatellite loci. The discrimination power of each microsatellite-locus was evaluated with 330 ‘artificial chimeras,’ made by mixing DNA from three different *S. pistillata* genotypes in pairwise combinations. In 68% of ‘artificial chimeras,’ the second genotype was detected if it constituted 5–30% of the chimera. Analyses of *S. pistillata* chimeras revealed that: (a) chimerism is a long-term state; (b) conspecifics were intermixed (not separate from one another); (c) disproportionate distribution of the conspecifics occurred; (d) cryptic chimerism (chimerism not detected via a given microsatellite) existed, alluding to the underestimation of chimerism in nature. Mixed chimerism may affect ecological/physiological outcomes for a chimera, especially in clonal organisms, and challenges the concept of individuality, affecting our understanding of the unit of selection.

## Introduction

Many biological studies assume that an organism, even a clonal one, is composed of a single genotype. This may not always be the case. Chimerism is the biological state of an organism harboring cells derived from at least two genetically distinct conspecifics^1^. Although chimerism occurs in a myriad of taxa^2^ within protists^3^, fungi^2,4^, plants^5^, algae^6,7^, sponges^8^, tunicates^9,10^, cnidarians^10–12^, crustaceans^13^, echinoderms^14^ and vertebrates, including humans^15^, knowledge about its ramification on the ecology and evolution of the organisms is sparse. Chimerism is a disparate phenomenon from genetic mosaicism, which is brought about by the presence of a somatic mutation in a subset of cells that differs from the inherited germline genome^16,17^. Yet, when studies find genetic differences within a single individual, they often attribute them to somatic mutations^4,18–22^. Chimerism challenges the concept of individuality and the unit of selection^23–25^, and highlights chimeric specific traits^26–28^. Chimerism can confer benefits to the chimeric individual^2,26,27^ such as enhanced survivorship^11^, increased growth rates^11,29^, enhanced reproductive output^6,30^, and stress resistance^30,31^. Conversely, costs to chimerism^3,27^ may include somatic and germ cell parasitism^21,26^, morphological disorders^26,29,32^, diseases^26^ and reproductive sterility^33^.

To address the importance of chimerism in a given species, including the role of the conspecifics in the ecology and physiology of the chimeric entity, it is imperative to understand how the different genotypes are distributed within the chimera. Chimeras may occur as: (a) mixed chimeras, where both partners are intermixed in the soma, in equal or different proportions. Various types of mixed chimerism were recorded in vertebrates, (further coined in humans as microchimerism)^15^, tunicates^28^, sponges^8^ and algae^34^, (b) sectorial chimeras, where allogeneic partners occupy territories within a single organism without being intermingled on the cellular level^18^, or (c) purged chimeras, where one of the partners is eventually purged from the soma, an outcome documented in tunicates^9^. Sampling methodologies, ethical and/or temporal challenges constrained the evaluation of, and conclusions about, spatial distribution within chimeras. For example, in many studies, as in vertebrates^15^, the whole organism was not investigated, or the spatial distributions within chimeras were followed for only a few days (sponges^8^).

Furthermore, whenever a chimeric entity gives rise to clones, the spatial distribution of the conspecific genotypes may affect the reproductive outcome and population structure, particularly in taxa that reproduce asexually and do not sequester the germ-line. Depending on the genotypic distribution within the chimera, the resulting clones may also be chimeras or include only one of the conspecific genotypes. Many hermatypic corals, the frame builders of coral reefs, can produce clones. In addition, the vast majority of hermatypic corals are colonial organisms, whereby, following settlement and metamorphosis of the larva (planula), the primary polyp, via budding, produces the coral colony. Hermatypic corals can exhibit chimerism^11,12,29,32,35,36^. Chimera establishment occurs in the early astogenetic stages^35,37^. One of the coral species capable of forming chimeras is the brooding coral *Stylophora pistillata*^11,30–32,37^, a species widely distributed in the Indo-Pacific and the Red Sea, where it is abundant on shallow coral reefs^38^. Since chimerism in *S. pistillata* is well established, the objectives of this study were to determine the spatial distribution of conspecific genotypes in up to 25-month old chimeras and to establish the detection threshold for chimeras utilizing microsatellite markers.

## Results

To establish microsatellite detection threshold, tissue samples were taken from three *S. pistillata* colonies (genotypes, SP1, SP2, SP3), situated ~ 4–6 km apart and 30 pairwise genotypic combinations were performed (called ‘artificial chimeras’; detailed in “Methods”, Fig. S1). We then studied the spatial distribution of genotypes within established *S. pistillata* chimeras obtained from kin and genetically non-related planulae. The majority of the chimeras were derived from kin planulae, and only a few arose from genetically non-related planulae. This outcome was a consequence of either mortality after fusion or rejection of conspecific spat (young settlers) forming the chimera in the early astogeny.

### Microsatellites’ discrimination power

We scored 30 pairwise genotypic combinations, which resulted in 330 ‘artificial’ chimera mixtures. The thresholds for detecting the least common partner in artificial chimeras depended on the genotypes compared (SP1 vs SP2, SP1 vs. SP3 or SP2 vs. SP3) and the microsatellite locus used (Table 1). In many instances, we detected the co-occurrence of both genotype peaks in the electropherogram, while in others, images with smaller peaks (ranging 100–500 fluorescence units) at expected bp sizes were visible (Table 1). Five scenarios (number of allele/locus at specific SP combinations) of SP3 vs. SP1 or SP2 were not fully resolved (Table 1). The combination SP3 > SP2 showed two alleles/locus (Stylo_72), preventing us from discriminating between the two genets (cryptic chimerism, chimerism not detected via a given microsatellite [CR-Chim]). SP2 > SP3 with microsatellite Stylo_72, failed to discern the least common genet. Additionally, in SP1 vs. SP3, microsatellite Stylo_55 was unable to disclose the two genets (Table 1). The co-occurrence of both conspecific genotypes in the ‘artificial chimera’ combinations was detected at different thresholds. In 28% of the ‘artificial chimera’ combinations, the least common genotype was detected even when it constituted only 5–10% of the genetic mixture. In 20% of the ‘artificial chimeras’, the proportion of the least common genotype had to be above 15–20% or 25–30% for it to be visualized. In another 20%, the threshold for detection of the less common genotype was when it constituted 35–40% of the ‘artificial chimera’ combination, and in 12% of the combinations, detection of the second genotype occurred at 45–50%. When considering the genotypic combinations with low fluorescence units, 5–10% thresholds for detecting the least common genotype were needed in 60% of the combinations, while 15–20% and 25–35% thresholds levels, each, were required in 20% of the combinations.

**Table 1:**
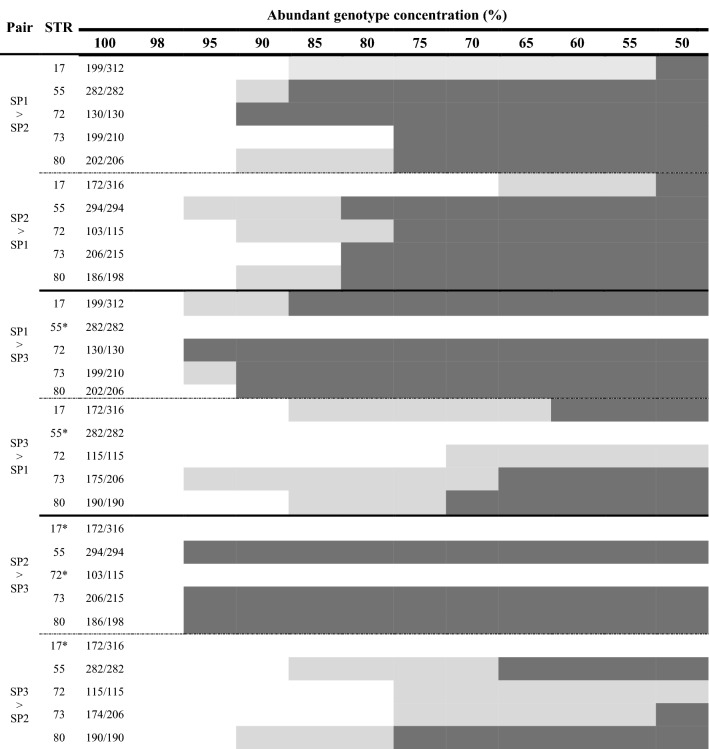
*Stylophora pistillata* ‘artificial chimeras:’ genet profiles and the detection thresholds of microsatellite loci. All possible pairwise combinations (Pair) of DNA mixtures of three *S. pistillata* genotypes (SP1, SP2, SP3), presented from 100% of the abundant genotype/0% second genotype to 50%/50% genotypic concentration ratio. Five microsatellites (STR, Stylo_n) are presented, with microsatellite allele sizes (bp) appearing in the 100% column. Dark-grey areas highlight DNA ratios where both genotypes were clearly detected by a specific microsatellite; light-grey areas highlight DNA ratios where the least common genotype was elucidated in the 100–500 fluorescence unit range. Asterisks denote non-resolutive microsatellites due to shared allele sizes in specific pairwise combinationns.

### Intra-organismal architecture of coral chimeras

**Figure 1:**
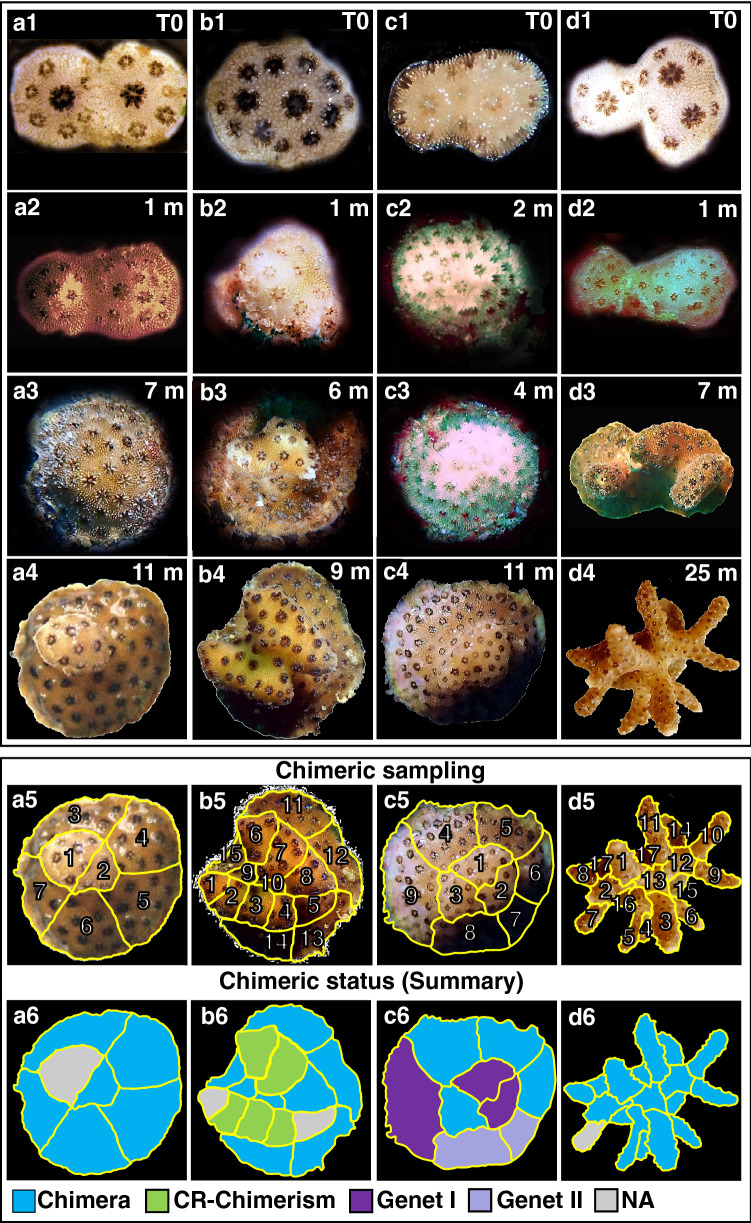
Chimerism and its detection in the coral *Stylophora pistillata*. Three bi-chimeras Chimera_33 (**a1**–**4**), Chimera_40 (**b1**–**4**), and Chimera_82 (**c1**–**4**) and a three-partner chimera, Chimera_43 (**d1**–**4**) are depicted. Chimeric sampling (**a**–**d5**) occurred 9–25 months post chimera formation (T0). The samples fragmented from each chimera were numbered. Chimeric status (**a**–**d6**) was determined based on microsatellite loci analyses. A colony fragment either exhibited a mixture of Genet I and II (Chimera), cryptic chimerism (CR-Chim), or only Genet I or Genet II. *NA* data not available for that fragment due to degraded DNA, lack of PCR amplification, or no resolution due to non-informative microsatellites.

Fusion between spat often started with a limited zone of continuous tissue/deposited calcium carbonate between the primary polyps (Fig. 1). In early astogenic stages following fusions, it was possible to morphologically distinguish between the founding partners (Fig. 1). After 2–6 months, the fusion of the partners progressed into a morphologically united coral colony where usually neither one of the partners could be discerned morphologically (Fig. 1). To reveal the presence and type of chimerism, microsatellite profiling analyses were performed on the nine chimeras (8 made of kin, 1 of genetically non-related offspring; cut to 154 fragments), sampled up to 25 months following fusion. To substantiate the chimeras, microsatellite profiling was also performed on the eight mother colonies (Table S1; A–H; 40 fragments), and two control colonies, non-chimeric colonies formed from planulae released at the same time as the planulae that formed chimeras (Fig. S2, Table S2; 14 fragments). The maternal colonies and the two 25-month-old control colonies were homogeneous for all eight microsatellite loci studied with no detectable mosaicism or chimerism (Table 2, Table S2). Out of the nine chimeras the microsatellite profile in four of these chimeras (Chimera_33, 40, 43 and 82) revealed the presence of a third allele in several fragments, expounding the chimeric status of these colonies (Fig. 1, Figs. S4, S5, S6, S7, Table 2). The microsatellite profiles of the other five chimeras contained only two alleles/locus (Fig. S3, Table S3), and were thus considered to consist of a single genotype (taking into account the discrimination power of microsatellites, as revealed from the ‘artificial chimeras’ results).

**Table 2:**
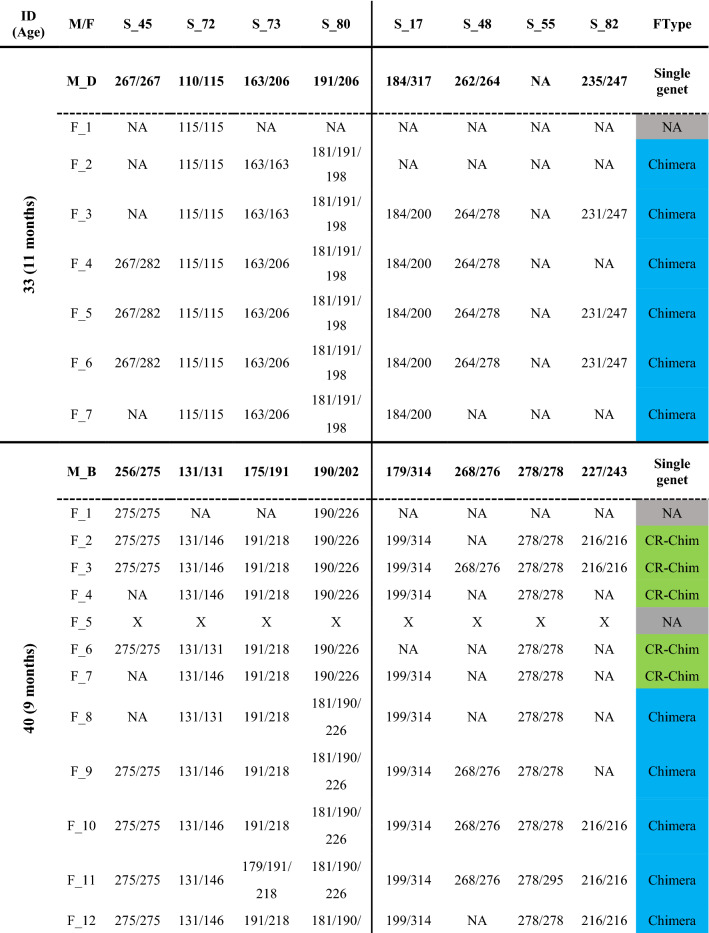

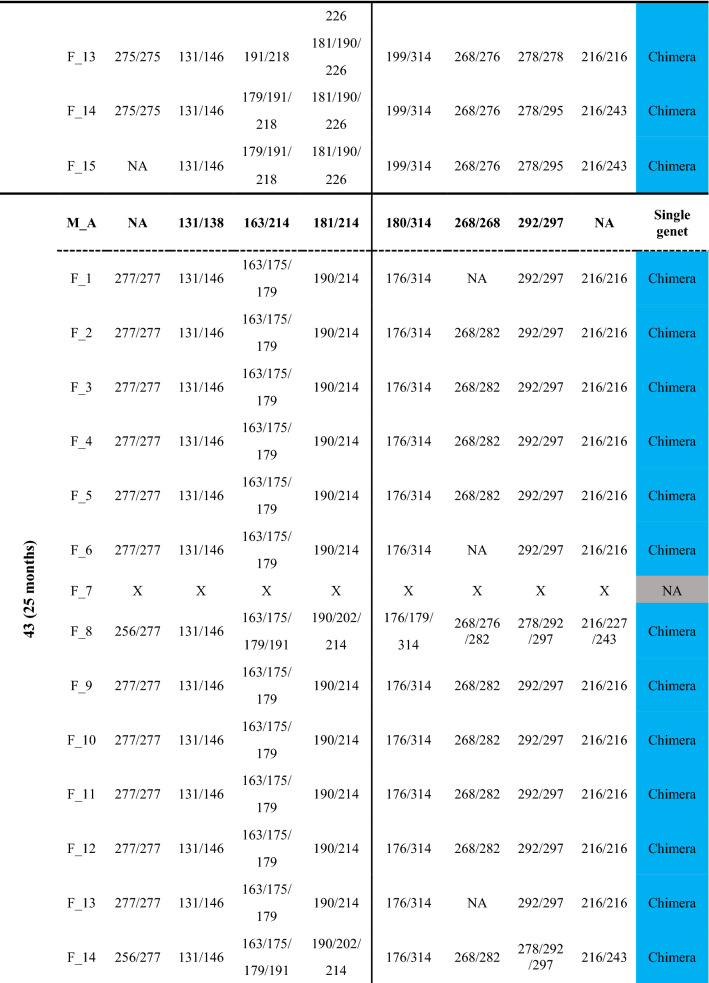

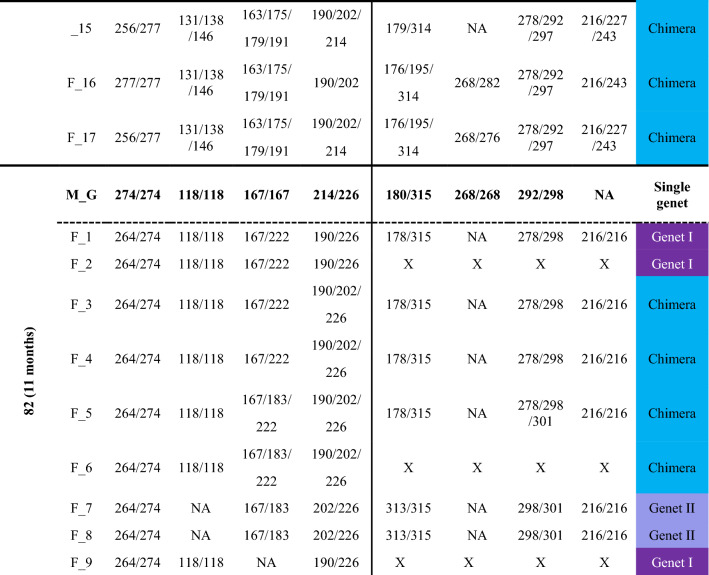
Microsatellite (S_n) allele sizes (bp) in *Stylophora pistillata* maternal colonies (M) and fragments (F) of the chimeric spat (ID). The classification of each fragment (F_n) as chimera, cryptic chimerism (CR-Chim), Genet I or Genet II, is provided in the fragment type (FType) column. The location of each fragment within a given chimera is presented in Fig. 1a–d5. *NA* data not available due to no visible peaks or weak peaks (< 100 fluorescence units) in the electropherogram; *X* fragments with degraded DNA.

Chimerism in the 11-month-old Chimera_33 was detected in 6 of the 7 fragments (one fragment yielded no DNA), demonstrating that chimerism existed nearly a year after the chimera formation (Fig. 1, Fig. S4; Table 2). Although the microsatellites were variable, their discerning power was not uniform. Microsatellite Stylo_80 (alleles 181/190/198) was the only polymorphic microsatellite locus that clearly showed chimerism in all fragments, unveiling the cryptic chimerism (CR-Chim) status in these fragments, as chimerism was not detected in some instances by other microsatellite loci. Microsatellite Stylo_73 showed homozygosity in fragments 2 and 3 (alleles 163/163), while fragments 4–7 showed the two alleles of the mother colony (163/206), microsatellite Stylo_72 was homozygotic on a mother colony’s single allele (115 bp) and microsatellite Stylo_55 did not successfully amplify any allele in either Chimera_33 or the maternal colony (Fig. S4, Table 2).

Chimera_40 was collected after 9 months in the nursery and showed chimerism in 12 of the 15 fragments (two fragments failed to amplify and one fragment was resolved as a single genet), where 4 of the 12 fragments were identified as CR-Chim (Fig. 1, Fig. S5, Table 2). Microsatellite Stylo_80 (181/190/226) detected the presence of chimerism in fragments 8–15, results supported by microsatellite Stylo_73 (179/191/218) in fragments 11, 14 and 15 (Fig. S5). Chimerism was further identified by microsatellite Stylo_80 in fragments 8–15 with cryptic chimeric status in fragment 7, that were assigned as chimeras by Stylo_72. As we did not detect fragments with single genets, fragments 2–4 and 6–7 were assigned as cryptic chimerism. Oddly, microsatellite Stylo_82 did not detect the mother allele in fragments 2, 3 and 10–13 (Table 2).

**Figure 2:**
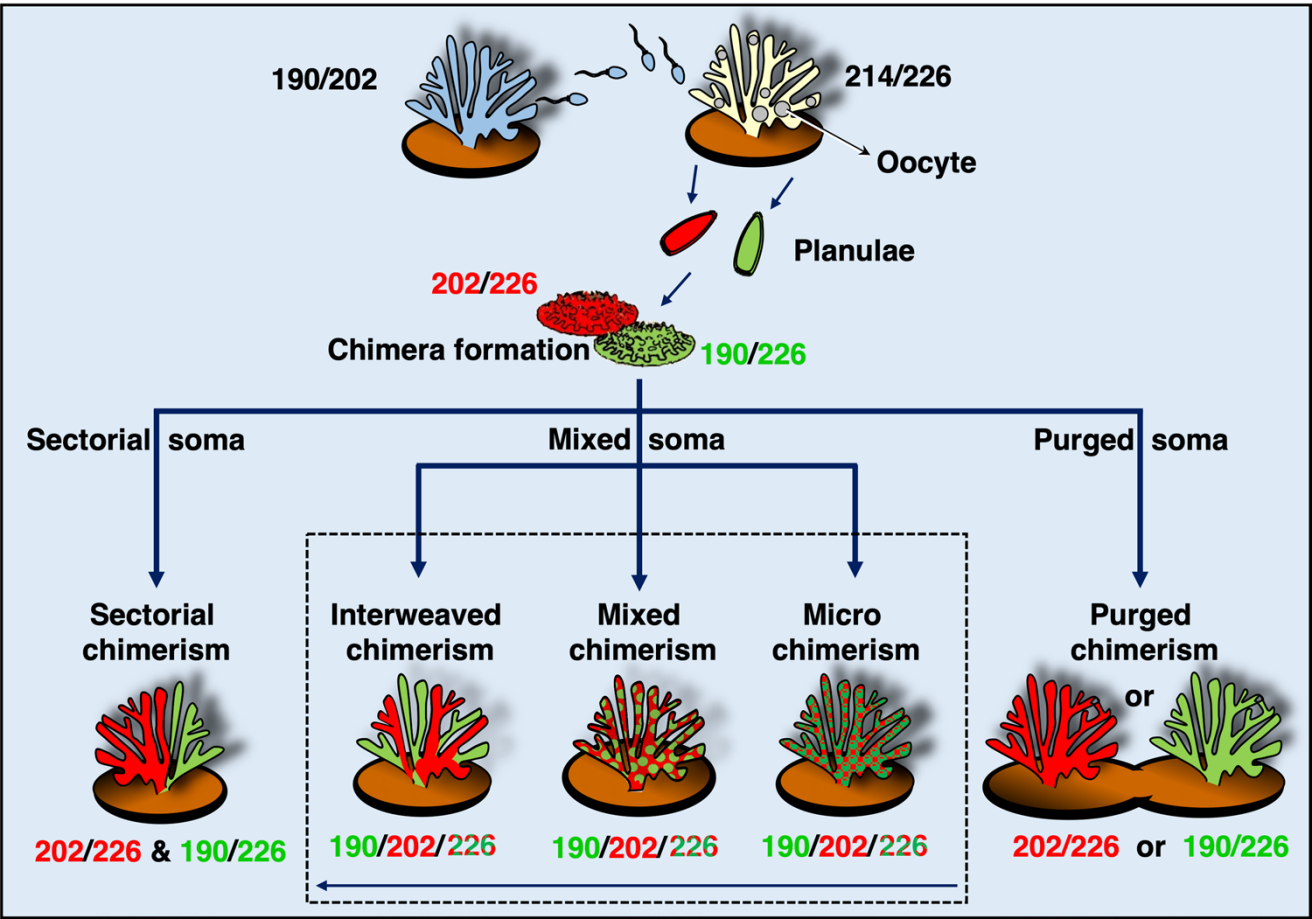
Chimera formation and potential outcomes in corals. Following allogeneic fusion of coral spats, three possible somatic outcomes may develop (*sectorial soma, mixed soma* or *purged soma*). *Sectorial soma*—genets are clearly segregated into distinct regions of the animal, without cellular intermingling, leading to sectorial chimerism. *Mixed soma*—genets are intermixed in three potential ways: (**a**) Interweaved chimerism—each genet dominates different colony fragments (i.e. branches or sectors of the coral colony); (**b**) mixed chimerism—cells of both genets are intermixed; (**c**) micro-chimerism—genets are confined to smaller colony areas (i.e. few polyps). The underlining arrow below the three scenarios projects the descending admixture levels of the genets along a continuum; *Purged soma*—a former chimera (purged chimerism) where a single genet overtakes the whole colony. The mixed soma scenarios further differ from the purged and sectorial soma by presenting chimeric statuses where 50% of the combined chimeric soma is of the shared allele (large ‘chimeric’ digits), and two unshared alleles (regular size digits), each distributed as 25% of the soma.

In the 11-month-old Chimera_82, the chimeric status was detected in 4 of the 8 fragments mainly by microsatellite Stylo_80 (alleles 190/202/226) in fragments 3–6, while fragments 1, 2 and 7–9 revealed two distinct genets, genet I (fragments 7, 8; alleles 202/206) and genet II (fragments 1, 2, 9; alleles 190/206, Fig. 1, Fig. S6, Table 2). Microsatellite Stylo_73 assigned chimerism to fragments 5 and 6 and microsatellite Stylo_55 for fragment 5 (Fig. S6, Table 2). Microsatellite Stylo_48 amplified neither allele in the chimera in contrast to the mother colony. Chimera_82 further exhibited an interweaved type of chimerism (Fig. 2). For example, the DNA in fragment number 8, located at the base of the colony was that of genet I, while in the adjacent fragment number 9, and in fragments 1 and 2, only genet II was detected. Conversely, fragments 3, 4, 5 and 6 contained both genets. This varied distribution suggests an uneven distribution of the soma from both genets in the chimera.

Multi-Chimera_43 was the results of a fusion between 3 spats. This chimera was processed at the age of 25 months. Chimerism was revealed in 16 of the 17 fragments (degraded DNA in fragment number 7), demonstrating that chimerism existed over 2 years after the chimera formation (Fig. 1, Fig. S7, Table 2). Microsatellite Stylo_73 revealed chimerism in all 16 fragments. Fragments 8, 14–17 showed 4 alleles (163/175/179/191 bp) representing only a single allele from the mother colony (allele 163), while fragments 1–6 and 9–13 showed 3 alleles (163/175/191 bp). Chimerism was also detected by microsatellite Stylo_72 in fragments 15–17 (131/138/146 bp), by microsatellite Stylo_80 in fragments 8, 13–14 and 17 (190/202/214 bp), by microsatellite Stylo_55 in fragments 8 and 14–17 and by microsatellite Stylo_82 in fragments 8, 15 and 17.

## Discussion

In colonial organisms, including corals, what constitutes an ‘individual’ is not straightforward, and chimerism further increases the complexity of the concepts of ‘individuality’ and the ‘unit of selection’^23–25^. Coral chimeras further challenge the prevailing tenet that a coral colony consists of genetically identical soma. By following coral chimeras from their inception, the most parsimonious explanation is that the within colony genotypic variation observed in the coral *Stylophora pistillata* arose from chimerism and not mosaicism because of (1) the visual observation of fusion of young spat, (2) the age (up to 25 months) of the sampled chimeras, ages where somatic mutations should not dominate the colony, and (3) the lack of mosaicism in control colonies of the same ages. We were able to address in these chimeras a key fundamental aspect for comprehending chimerism, and that is the spatial distribution of conspecifics within a chimera (the conspecific landscape) that may affect the physiology and ecology of the chimeric coral colony. Furthermore, the ramifications of the spatial distribution are substantial and far-reaching. For example, in addition to sexual reproduction, most coral species can asexually produce stand-alone daughter colonies via colony fragmentation^39^ that are considered clonemates. If a chimera asexually reproduces, the distribution of the conspecific genotypes may affect the genetic makeup of the new colonies. These colonies may indeed be clonemates, genetically identical to the original coral chimeric colony. Alternatively, the new colonies may only include one or a subset of the conspecific genotypes. Although, our understanding of the occurrence of chimerism in corals is still limited, we suggest that, depending on frequency of coral chimerism, either scenario will affect the genetic composition of coral larval pools, the population structure of the coral species, and subsequently the coral reef.

When a chimera forms, three outcomes may occur (Fig. 2). The first is a purged chimerism, where the chimera may not persist, resulting in a coral colony composed of a single genotype. Our study demonstrates that in *S. pistillata,* chimerism is not transient. Unlike purged chimerism in colonial tunicates^9^, *S. pistillata* exhibited long-lasting chimerism, maintaining the chimeric state up to 25 months following fusion at which point the colonies were sampled (yet, it should be noted that chimerism was not detected in 5/9 colonies, a result that may indicate either a purging process or a state of micro-chimerism). Furthermore, considering the documented chimerism in wild adult coral colonies^12^, we conclude that chimerism persists in *S. pistillata*, potentially for the entire lifespan of coral colonies. Within lasting chimeras, the conspecific genotypes may be spatially distributed in two main ways. The conspecifics may occur side by side in the coral colony without the genotypes mixing, a sectorial soma scenario (Fig. 2). Conversely, the conspecifics may be intermixed, a mixed soma scenario, with various degrees of intermixing (Fig. 2). Although a prior study assumed that coral chimeras exhibit sectorial chimerism, Fig. 1a in^18^, whether the conspecific genotypes within a chimeric colony are sectorially distributed or mixed can only be determined via spatial sampling.

Our extensive within-colony sampling revealed that *S. pistillata* chimeras actually displayed mixed soma chimerism (Fig. 1, Figs. S4, S5, S6, S7). Within mixed soma, three levels of intermixing can theoretically occur, micro-chimerism, where genets are well intermixed; mixed chimerism, where genets are confined to smaller colonial areas, such as a few polyps; and interweaved chimerism, where each genet dominates the soma of large fragments, such as branches or colonial sectors (Fig. 2). The ability to differentiate between the three levels of intermixing in mixed soma will be feasible as molecular techniques advance. Although microsatellites are currently the best method to discern partners within chimeras, this approach, as any approach, has methodological aspects that need to be considered when interpreting the data. First, if both conspecifics share alleles in a locus (e.g. allele 226 in Fig. 2) then in the chimeric sample there will be, theoretically, two times more of one allele (e.g. 226) than either one of the other alleles (e.g. 190 or 202). These different DNA concentrations may lead to an over visualization of the shared allele concurrent with an under visualization of the other alleles. Alongside such a scenario, because PCR is affected by the different DNA concentrations, it is possible that, even though DNA of one chimeric partner is present, its amplification is out competed in the PCR reaction. For example, in Chimera_40, 8/14 fragments exhibited chimerism. This status, however, was not visualized for some of the remaining fragments, likely due to shared alleles between the maternal and the sperm donor/s or due to microsatellite detection thresholds (Fig. 1b6, Fig. S5). When such a situation arose, we termed this phenomenon as a cryptic chimerism.

Our study also demonstrates the challenges of investigating chimerism and the conspecific landscape. To comprehend chimerism, one needs to detect the existence of chimeras. As our findings show, after the initial fusion between the conspecific coral spats, visually it is impossible to identify the location of each of the genotypes in the chimeric colony (Fig. 1, Figs. S4, S5, S6, S7). Thus, genetic techniques are necessary to not only identify the existence of coral chimeras but to also decipher the spatial distribution of the genotypes within a chimera. Since chimeras involve conspecifics, evaluating microsatellites is currently the most effective approach. Although next generation sequencing (NGS) methodologies can detect even fine genotypic variations within a coral colony e.g.^40,41^, as they are currently employed, these methodologies cannot better distinguish the prevalence of one genotype over another, an important aspect in interpretation of genotypic spatial distribution. While the use of additional microsatellites would improve detection, the 8 microsatellite loci used in the study are the only highly polymorphic microsatellites available. Nevertheless, these microsatellites provided excellent differentiation power between the coral genotypes in our artificially formed chimeras.

Microsatellites are currently the best method to discern partners within chimeras and their spatial distribution, but this technique has its detection limits^42^. Based on the 330 ‘artificial chimeras’ that we produced in this study, microsatellites started to disclose the least common genotype at a threshold ratio above 5–25% (Table 1). In 12% of the pairwise conspecific combinations, the least common genotype appeared within the fluorescence electropherogram in very low range of fluorescence units and was definitively detected only when it constituted 45–50% of the DNA ratio (Table 1). Even though the *S. pistillata* colonies used to produce the ‘artificial chimeras’ in this study were ~ 4–6 km apart, they shared some microsatellite alleles, potentially revealing coral population connectivity in the region, and/or diminished allelic polymorphism. Furthermore, most probably due to some methodological constraints (e.g., low allele polymorphism, high percentages of homozygote loci, genotypic ratios below the detected levels of the microsatellites), we clearly elucidated chimerism in four of the nine established chimeras. Our findings, from both the ‘artificial’ and established chimeras, demonstrate that many chimeric states may stay undisclosed, similar to what occurs in human micro-chimerism^15^ and in blood-transplanted patients^42^, where standardization of methods has been lacking, and difficulties in detecting chimerism necessitate special methodological attention^43^.

Low detection of chimerism is further compounded if not enough replicates of a specific colony are sampled. Limited within colony sampling, usually 1–4 tissue samples/coral colony^18,20,21,44^ may further contribute to underestimation of the numbers of natural chimeras. In addition, when the distribution of the conspecific genotypes in a chimera is not uniform, as we found in our study, it may further lead to the underestimation of chimerism brought about by collecting only a couple of samples per colony. When examining multiple samples per colony may not be experimentally feasible, or cost prohibitive to process on a large scale, such methodological constraints should not lead to the dismissal of chimerism as a potentially important aspect to a given coral species’ physiology and ecology. Increasing sampling within a coral colony, and even sampling an entire small coral colony, as performed in our study, could affect the interpretation of the abundance of chimeras on coral reefs.

Even when different tissue samples from a single colony diverge in a significant portrayal of loci or alleles, the interpretation of these genetic differences is debated. Hitherto, the literature on marine organisms assigned mosaicism (somatic mutations) as the explanation for almost all documented intra-colony genetic variations^18–21,34,45^. This notion was further framed by the statement that “a chimera had to include genotypes that differed more than 60% in their cluster assignment probability” obtained from the Bayesian clustering analysis in STRUCTURE^21^. Yet, a study on chimeras of the hydrozoan *Ectopleura larynx*^41^ suggests that homozygote loci may reduce the power of the calculations for multiple distinct genotypes, thus leading to erroneous attribution to mosaicism. In addition, if kin produce chimeras, the proportion of chimeras in which the conspecifics exhibit homozygotic loci may be even higher than for non-kin. Furthermore, our comprehensive sampling of *S. pistillata* colonies known to have originated from one genotype did not detect genetic variation, reiterating that somatic mutation may not be the most parsimonious explanation for genetic variation within a coral colony. Therefore, when within coral colony genotypic variability occurs, it would be beneficial to consider chimerism, and the potential ramifications of chimerism, and not dismiss the finding as a localized, non-consequential, mutation.

Understanding chimerism may affect the comprehension and interpretation of coral colonies, coral population structure, and coral reefs in general. Further, the spatial distribution of conspecifics within the chimera adds relevancy when elucidating the costs and benefits of chimerism^2,11,27,29,46^, including somatic and germ cell parasitism^2^ and in understanding the ecological and evolutionary traits associated with chimerism^30,31,47^. Examples are the enhanced variations recorded in coral chimeras with chimeric traits as compared to non-chimeric individuals e.g., survivorship^48^; or the roles in pathogenesis of fetal cells microchimerism located in various female internal organs, e.g.^49^. The variation in genetic constituency in chimeras, led to the suggestion of targeting chimeric states in corals as an applied tool for fuelling selection and rapid adaptation in a changing world and under global climate impacts^24^.

## Conclusions

Chimerism can be deeply integrated in the life history of species, as well as in processes such as the allorecognition machinery^50^ and alloimmune maturation^37^. Our study, while elucidating long term chimerism and complexities of mixed soma in the coral *S. pistillata*, further illustrates that widely used genetic sampling strategies may miss out this diverse complexity. Coral chimerism may be more common than currently thought and may play an important role in the ecology and physiology of the coral species in which it occurs. Clearly, this field necessitates more detailed studies on the possible dynamic statuses of chimerism in corals and other organisms and requires the advent of specific molecular techniques that do not rely on pre-set assumptions. Although the existence of chimerism in corals has been documented for almost 120 years^51^, chimerism has not been extensively studied, often only mentioned in passing, and relegated as an oddity^12,21^. Accepting the existence of chimerism may drive scientists to not only distinguish different genomes within single ‘individuals’ but may further lead to more comprehensive understanding of the ecological and evolutionary perspectives of this phenomenon.

## Methods

### Experimental outline

This study included four steps: (1) maternal colony selection and sampling, (2) planula collections and chimera formation, (3) maintenance of chimera and control colonies, and (4) fragmentation of chimeric colonies and genotyping of chimeric fragments, controls and maternal colonies.

1. Eight gravid maternal (mother) colonies of the brooding coral species *Stylophora pistillata* were haphazardly chosen as larval (planula) donors. These colonies were located at 6–7 m depth in front of the Interuniversity Institute for Marine Sciences in Eilat, Gulf of Eilat, Red Sea (29° 30′ 04″ N; 34° 55′ 02″ E). Branch tips were randomly clipped for subsequent DNA analysis.
2. In corals, chimerism exclusively develops within a window of time in the early astogenetic stages^35,37,52^, necessitating working on planulae and/or spat (young settlers). Planulae were collected from the mother colonies using planula traps that were deployed at sunset and collected about 12 h thereafter (protocols in^53,54^). Planulae from the same maternal colony (kin), collected on the same night, were put in the same Petri dishes (110/17 mm) that were covered with translucent tracing paper (Graphic Vision Media), preconditioned in ex-situ tanks for 3 months. In these dishes, planulae settled and metamorphosed into primary polyps. Adjacent spats, whose tissues came into direct contact, often fused, forming chimeras. In other Petri dishes, genetically non-related planulae (from different maternal colonies) were manually put into proximity of each other, leading some to fuse and form genetically non-related chimeras.
3. Chimeric colonies were transferred to a mid-water floating coral nursery (suspended at 10–12 m depth over 25 m sea bottom, 29° 32′ 34″ N; 34° 58′ 24″ E) where they were kept until collections at 9–25 months old.
4. Each young colony was fragmented into 7–17 fragments. Samples from the chimeric fragments and from non-chimeric young colonies and maternal colonies were genotyped, and microsatellite analyses were conducted.

### Chimera formation

During 15 days post collections, *S. pistillata* planulae naturally settled in aggregates of two or more spats on the preconditioned translucid paper substrate, in *ex-situ* tanks with natural flowing seawater. Adjacent spats, whose tissues came into direct contact, often fused, forming chimeras (Fig. 1a1–d1, Figs. S4, S5, S6, S7). In other Petri dishes, genetically non-related planulae (from different maternal colonies) were manually put into proximity of each other, leading some to fuse and form genetically non-related chimeras. Chimeras were detached from the petri dishes, glued on plastic pins^55^ and deployed at the mid-water floating nursery (in-situ), covered by perforated plastic cages (3 × 3 cm mesh) that allowed water exchange and light transfer while eliminating fish grazing. In total, 56 kin and 36 genetically non-related bi-chimeras (two genets/chimera) and 26 kin and 4 genetically non-related multi-chimeras (more than two genets/chimeras) were deployed onto the mid-water floating nursery. The chimeras were cleaned and monitored every 3–5 months until sampling.

### Sampling

Samples for DNA analysis were collected from three sources: (1) Maternal colonies (n = 8). Tip fragments (~ 1.5 cm, 5 tips/colony) were haphazardly clipped off from different parts of each mother colony, brought to the laboratory where the upper ~ 0.5 cm of the tips, considered as sexually sterile zones in gravid coral colonies^56^, were sampled using a side cutting plier cleaned, after each cut, with a flame to avoid cross contamination. (2) Chimeras, collected from the coral nursery at different ages (9–25 months old), brought to the laboratory, photographed and each sectioned into 7–17 fragments (6–26 polyps/fragment) using a fine electrician cutter (Fig. 1a5–d5). Different chimeric ages enabled investigating the spatial distribution of genotypes within chimeras at different astogeny stages and morphological complexity. (3) Control (non-chimeric) colonies (n = 2, 25-months-old), originating from the same planula cohorts and raised under the same conditions as the coral chimeras, were fragmented and analysed for microsatellite profiling in order to evaluate the possibility of mosaicism^57^, the outcome of somatic mutations.

### DNA extraction and PCR amplification

Total genomic DNA from the *S. pistillata* chimeras, maternal colonies and control colonies was extracted from their respective tissue samples using phenol chloroform methodology^58^ with modifications^59^. The extracted DNA was amplified in multiple different PCR reactions with primers for 8 previously published^60^ microsatellite loci: Stylo_17, Stylo,_45, Stylo_48, Stylo_55, Stylo_72, Stylo_73, Stylo_80, Stylo_82. These microsatellites were chosen because they exhibited high: (a) number of alleles/locus, (b) observed heterozygosity, and (c) nucleotide motif length^61,62^.

PCR was performed in 20 µL total solution with 4–16 ng of extracted DNA (1–4 μL from 1:10 solution), 0.1 µM of each primer, 1 × of TIANGEN MasterMix (0.1 U/μL Taq Polymerase, 500 μM dNTP each, 20 mM Tris–HCl, pH = 8.3, 100 mM KCl, 2 mM MgCl_2_, stabilizer and enhancer), with double distilled water (DDW) to reach the reaction volume. The PCR thermal cycling protocol followed that of Banguera-Hinestroza et al.^60^, with number of cycles increased to 30.

All PCR reactions included at least 1 negative and several positive controls, depending on the microsatellite used. To further verify conformity of results, ~ 7% of the samples were randomly selected, independently re-amplified and re-sequenced for two haphazardly chosen microsatellite loci. Since the re-sampled 7% showed minor differences to the prior amplifications (intensity of the peaks but not size), there was no need to re-sample further. Negative reactions always resulted in no bands. Positive controls sometimes did not amplify, necessitating a PCR reaction re-run with a new primer solution which then led to positive amplifications. Samples whose DNA did not amplify or did not sequence successfully were re-processed at least 5 times. Positive PCR products (verified by the presence of the right size bands in agarose gels) were sent to the Rambam Health Care Campus, Israel for capillary electrophoresis analysis (Applied Biosystems 3500), using PET, FAM-6, NED and VIC as the fluorescent probes.

### Microsatellite’s discrimination power and interpretation

DNA was extracted from tissues of three *S. pistillata* colonies (SP1, SP2, SP3; separated by ~ 4–6 km), quantified using Qubit 2.0 Fluorometer (Life Technologies), diluted in DDW to a concentration of 53 ng/μL, and further diluted in DDW (1:25, stock DNA). DNA pairwise combinations (SP1 vs SP2; SP1 vs SP3 and SP2 vs SP3) at a gradient of 11 DNA concentrations (2–98%, Fig. S1), produced 330 ‘artificial chimeras’ each representing a different genotypic ratio. PCR amplifications and microsatellite analysis were performed as above. This analysis provided information on microsatellite detectable thresholds alongside disclosing chimerism with microsatellites even in cases of one shared allele/locus between genets. Any *S. pistillata* fragment whose microsatellite profile contained 3 or 4 alleles/locus was considered a chimera. Yet, it is also possible to have bi-chimeras where the sperm donor shares one or two alleles with the maternal inherited alleles, resulting in a single allele/locus. Thus, cases where fragments from a chimera (depicted by the presence of 3–4 alleles in at least one fragment) revealed both homozygotic and bi-allele heterozygotic status, without a clear separation of the two genets in at least two fragments (an example: Fig. 1c6, S6 Stylo_73), were termed as cryptic chimerism (chimerism not detected via a given microsatellite; CR-Chim). The genotypes revealed by the microsatellite markers, analysed with Geneious 11.1.4 software, were independently scored by two people and all unclear cases were re-analysed by a third person.

## Supplementary Information

Supplementary Information.
