# Publisher Correction: Spatial distribution of conspecific genotypes within chimeras of the branching coral *Stylophora pistillata*

**DOI:** 10.1038/s41598-021-03463-w

**Published:** 2021-12-08

**Authors:** Gabriele Guerrini, Dor Shefy, Jacob Douek, Nadav Shashar, Tamar L. Goulet, Baruch Rinkevich

**Affiliations:** 1 Israel Oceanography and Limnological Research, National Institute, of Oceanography, Tel-Shikmona, P.O. Box 9753, 3109701 Haifa, Israel; 2 Department of Life Sciences, Eilat Campus, Ben Gurion University of the Negev, Eilat, Israel; 3 The Interuniversity Institute for Marine Science, 88000 Eilat, Israel; 4 Department of Biology, University of Mississippi, P.O. Box 1848, University, MS 38677-1848 USA

Correction to: *Scientific Reports* 10.1038/s41598-021-00981-5, published online 19 November 2021

The original version of this Article contained an error in the Received date which was incorrectly given as 17 March 2021. The correct date is 06 August 2020.

The original Article has been corrected.

