# Supplementary material for: Spatial distribution of conspecific genotypes within chimeras of the branching coral Stylophora pistillata
Source: Sci Rep. 2021 Nov 19;11:22554. doi: 10.1038/s41598-021-00981-5 (PMC8604976; doi:10.1038/s41598-021-00981-5)
Supplement: Supplementary file 1 — Supplementary Information. [file 41598_2021_981_MOESM1_ESM.docx]

**Supplementary Information**

**Spatial distribution of conspecific genotypes within chimeras of the branching coral *Stylophora pistillata***

Gabriele Guerrini^1,2^, Dor Shefy^1,2,3^, Jacob Douek^1^, Nadav Shashar^2^, Tamar L. Goulet^4*^, Baruch Rinkevich^1^

^1^ Israel Oceanography and Limnological Research, National Institute of Oceanography, Tel-Shikmona, P.O. Box 9753, 3109701 Haifa, Israel

^2^ Marine Biology and Biotechnology Program, Department of Life Sciences, Ben- Gurion University of the Negev Eilat Campus, Beer-Sheva 84105, Israel.

^3^ The Interuniversity Institute for Marine Science, 88000 Eilat, Israel

^4^ Department of Biology, University of Mississippi, P.O. Box 1848, University, MS 38677-1848, USA


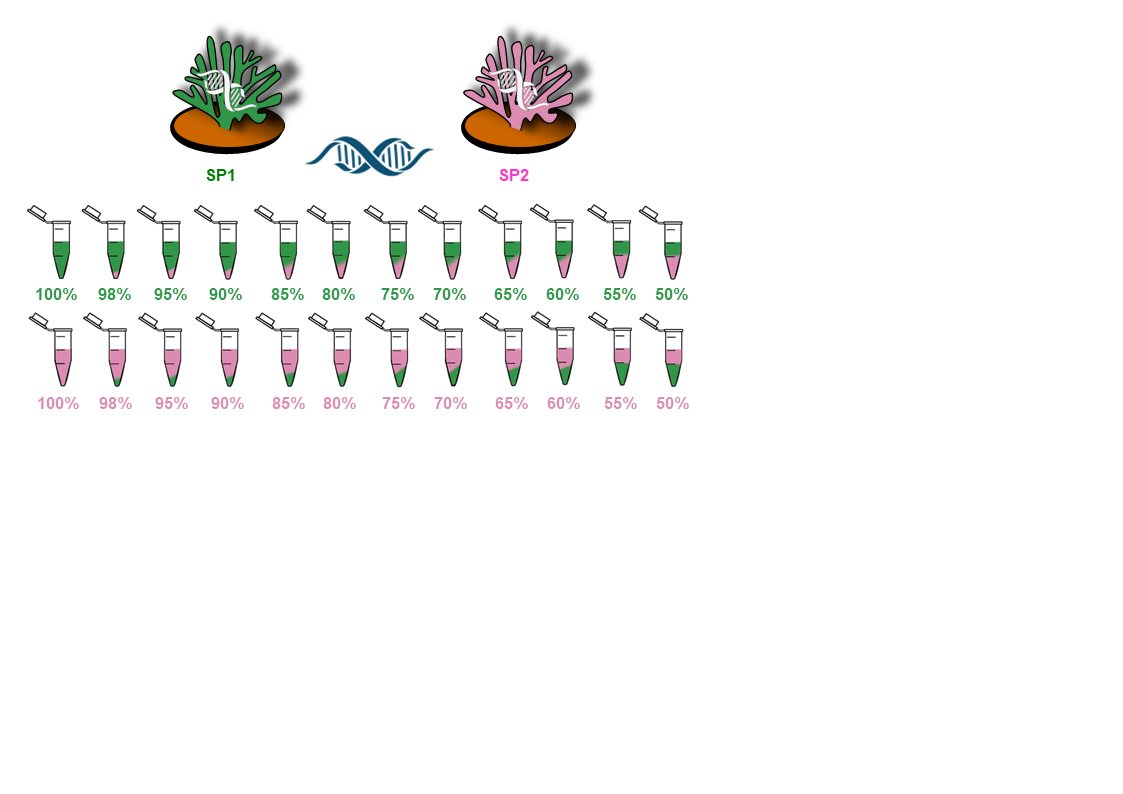


**Supplementary Figure S1** **A schematic illustration for the artificial chimeras’ set-up.** The picture exemplifies the case of a pairwise combination (SP1 vs SP2) with 11 descending DNA concentration ratios aiming to reveal pairwise combination thresholds for the positive identification of the less common genet in the chimera.


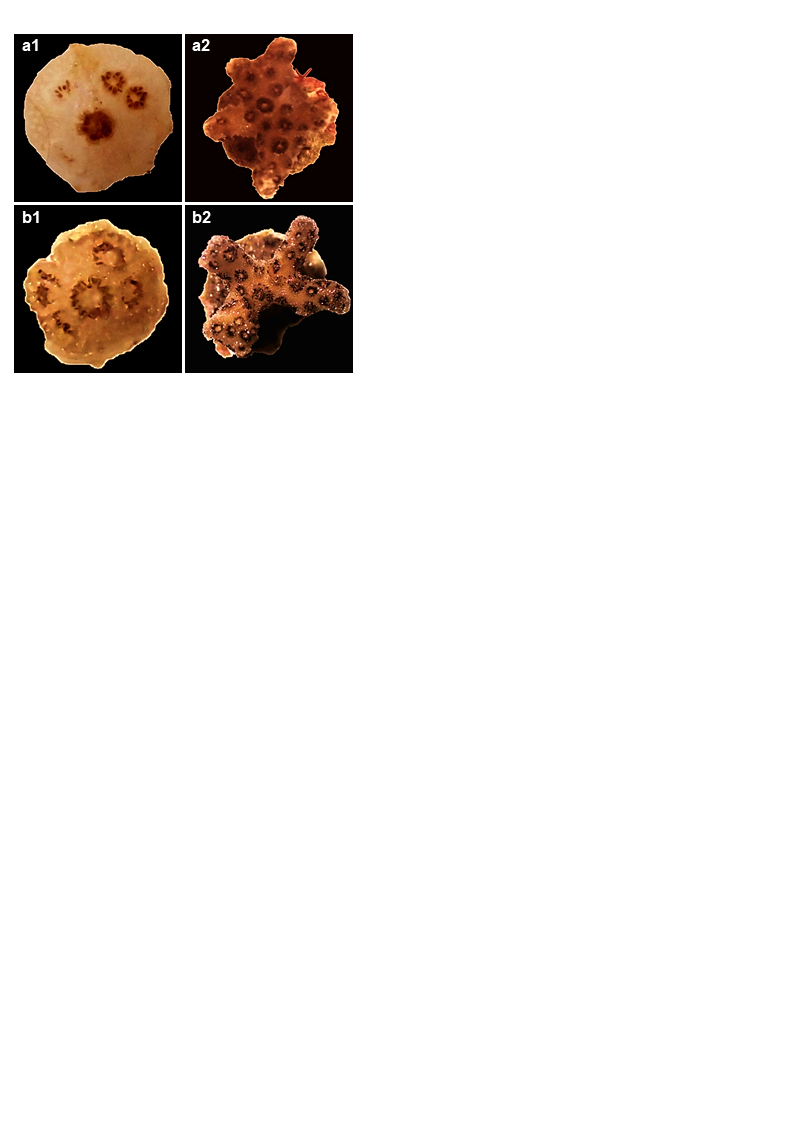


Supplementary Figure S2 Images of two *Stylophora pistillata* single genotype colonies from spat to colony stage. The two control colonies (a and b) used in the experiment photographed when they were spats (a1 and b1) and 25 month old colonies (a2 and b2).

Supplementary Figure S3 The five initially chimeric *Stylophora pistillata* colonies that were not elucidated as chimeras by the microsatellites. Documentation of fusions at onset (a1-d1) made with genetically non-related spat (a) and kin spats (b-d). The established chimeras when they were sacrificed: (a2) Chimera_03, 2-years; (b2) Chimera_44, 13-months; (c2) Chimera_49, 2 years; (d2) Chimera_74, 2-years; (e) Chimera_101, sampled at 11-months (fusion photo not available).


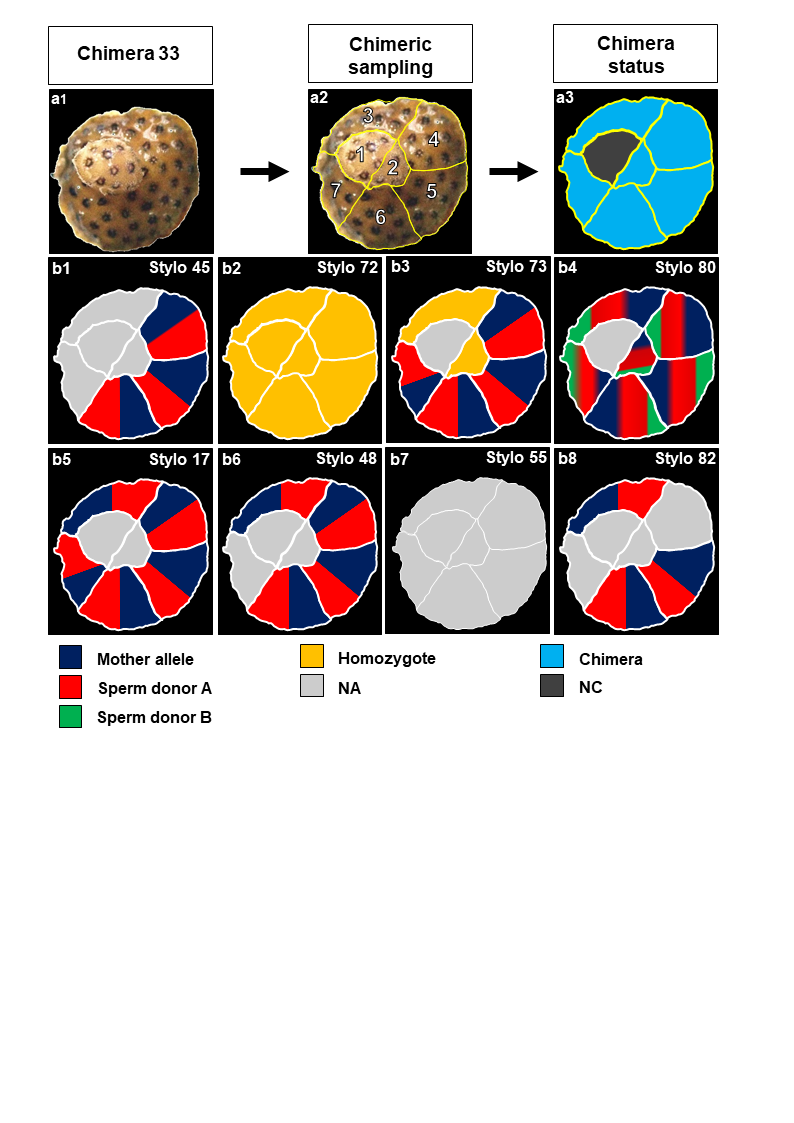


Supplementary Figure S4 Chimeric status for the entirely fragmented *Stylophora pistillata* Chimera_33, determined with 8 microsatellite loci on 7 fragments. A photograph of the chimera on sampling day (a1), and the numbered fragments sampled (a2). The overall chimeric status (a3) based on the composite results from the 8 microsatellite loci (Stylo_n, b1-b8). The microsatellites revealed allele sizes in the fragments that corresponded to either the mother colony allele (dark blue), a homozygotic state with sperm donor/s of the same allele size as the mother colony (yellow), different allele sizes derived from sperm donors ‘A’ or ‘B’ (red and green, respectively), and chimerism within a fragment (light blue). NA = data not available due to degraded or PCR failures or small peaks (<100 fluorescence units); NC = nonconclusive due to presence of several NAs and not informative microsatellites.


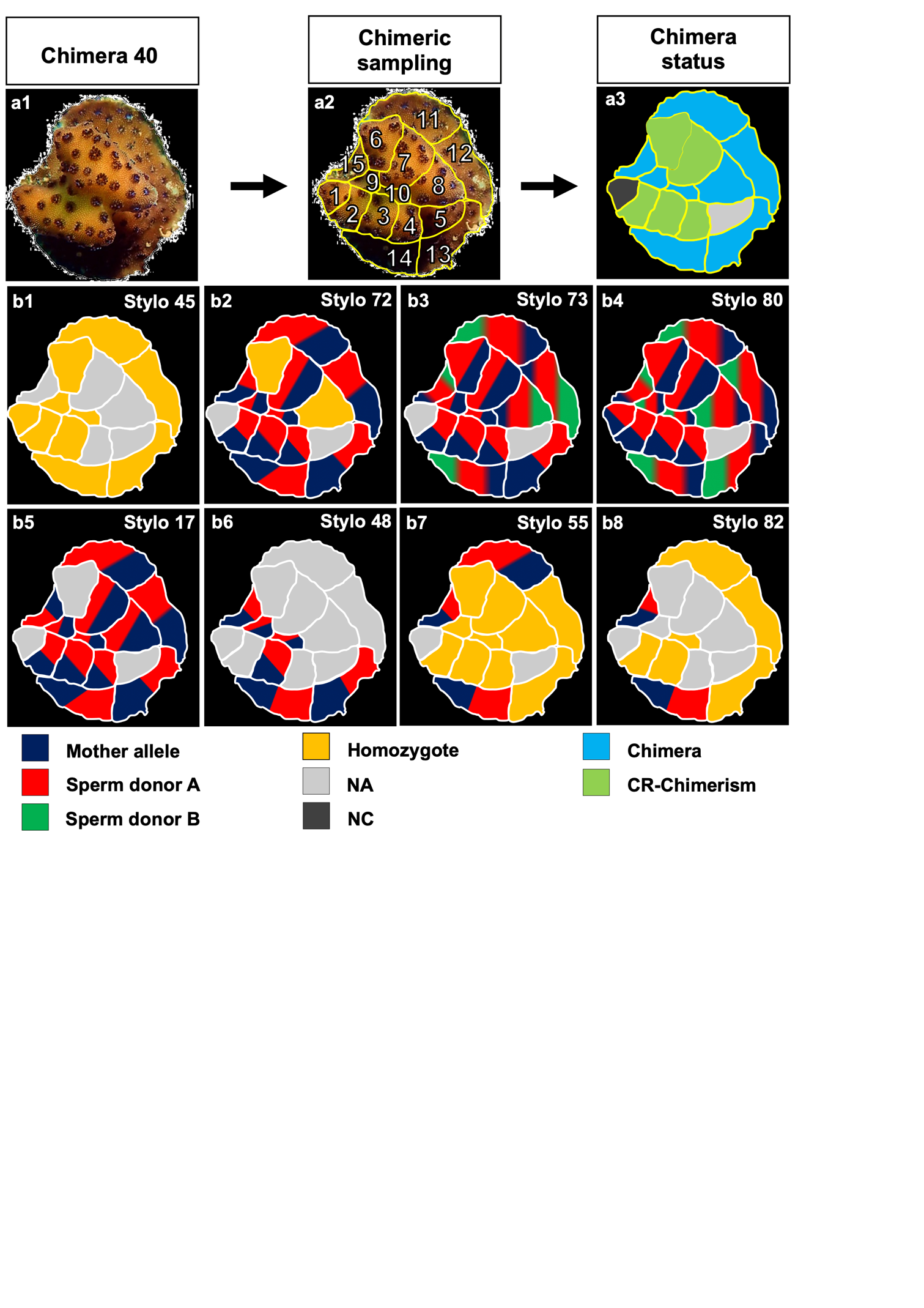


Supplementary Figure S5 Chimeric status for the entirely fragmented *Stylophora pistillata* Chimera_40, determined with 8 microsatellite loci on 14 fragments A photograph of the chimera on sampling day (a1), and the numbered fragments sampled (a2). The overall chimeric status (a3) based on the composite results from the 8 microsatellite loci (Stylo­­_n, b1-b8). The microsatellites revealed allele sizes in the fragments that corresponded to either the mother colony allele (dark blue); a homozygotic state with sperm donor/s of the same allele size as the mother colony (yellow), different allele sizes derived from sperm donors ‘A’ or ‘B’ (red and green, respectively), chimerism within a fragment (light blue), a cryptic chimera within the fragment (CR-Chim, light green). NA = data not available due to degraded or PCR failures or small peaks (<100 fluorescence units); NC = nonconclusive due to presence of several NAs and not informative microsatellites.


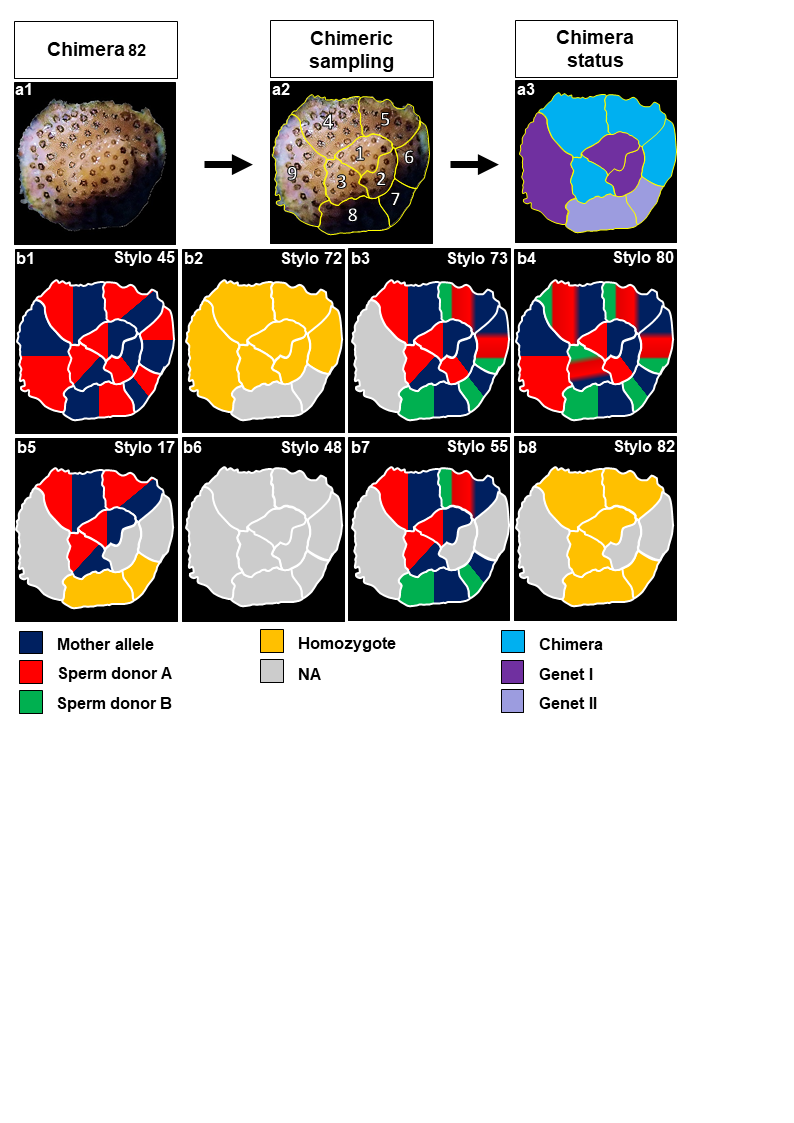


Supplementary Figure S6 Chimeric status for the entirely fragmented *Stylophora pistillata* Chimera_82, determined with 8 microsatellite loci on 9 fragments. A photograph of the chimera on sampling day (a1), and the numbered fragments sampled (a2). The overall chimeric status (a3) based on the composite results from the 8 microsatellite loci (Stylo­­_n, b1-b8). The microsatellites revealed allele sizes in the fragments that corresponded to either the mother colony allele (dark blue); a homozygotic state with sperm donor/s of the same allele size as the mother colony (yellow), different allele sizes derived from sperm donors ‘A’ or ‘B’ (red and green, respectively), chimerism within a fragment (light blue), and when only one of the genets was detected (Genet I or Genet II, purple and light purple, respectively). NA = data not available due to degraded or PCR failures or small peaks (<100 fluorescence units).


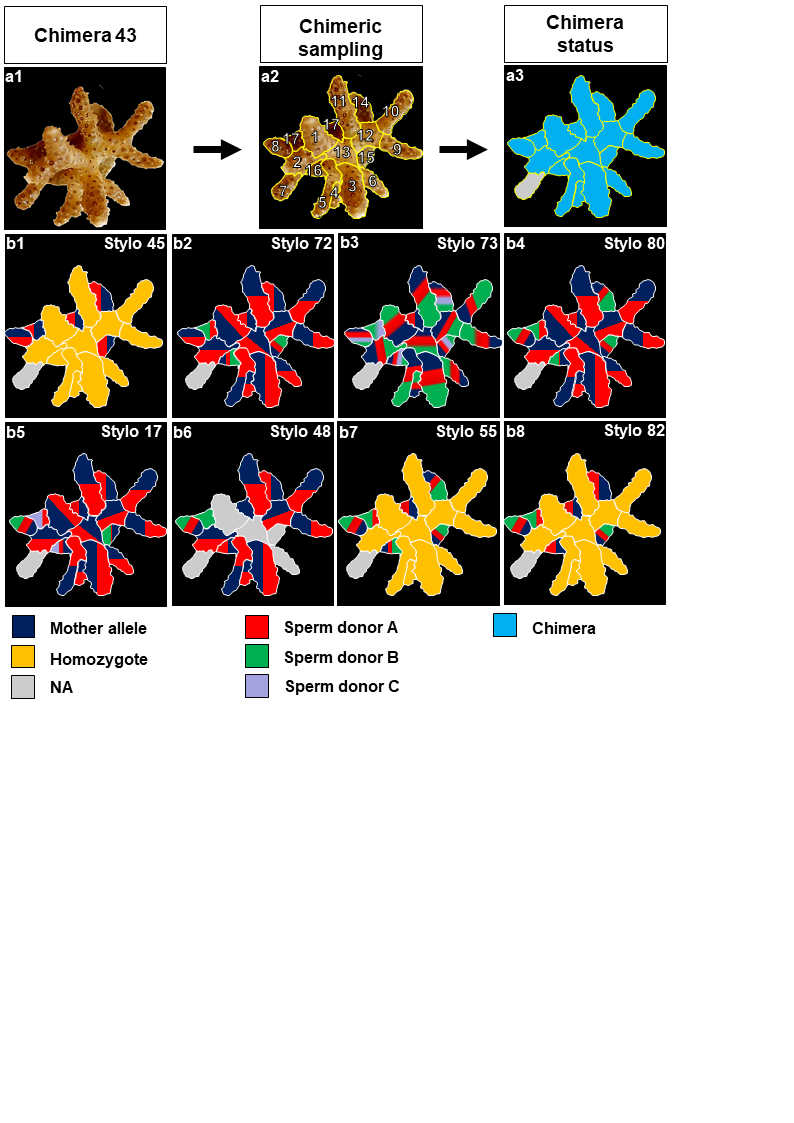


Supplementary Figure S7 Chimeric status for the entirely fragmented *Stylophora pistillata* Chimera_43, determined with 8 microsatellite loci on 16 fragments. A photograph of the chimera on sampling day (a1), and the numbered fragments sampled (a2). The overall chimeric status (a3) based on the composite results from the 8 microsatellite loci (Stylo­­_n, b1-b8). The microsatellites revealed allele sizes in the fragments that corresponded to either, the mother colony allele (dark blue), a homozygotic state with sperm donor/s of the same allele size as the mother colony (yellow), different allele sizes derived from sperm donors ‘A’, ‘B’ or ‘C’ (red, green and purple, respectively), and, chimerism within a fragment (light blue). NA = data not available due to degraded or PCR failures or small peaks (<100 fluorescence units).

Supplementary Table S1. Microsatellite allele sizes (base pair) in *Stylophora pistillata* mother colonies (A-H). All the samples from the same mother colony contained the same microsatellite alleles. NA = microsatellites that did not work. (*) refers to a mutation in the specific allele in a spat used to create chimera_49. Mother_D showed two additional alleles (103 and 134bp) in two different branch tips, respectively, likely a result of a developed planula larva within the maternal tissue.

| **Mother colony** | **GI** | | | | | | | | **GII** | | |  |
| --- | --- | --- | --- | --- | --- | --- | --- | --- | --- | --- | --- | --- |
|  | **Stylo_45**  **PET** | **Stylo_72**  **FAM-6** | **Stylo_73**  **NED** | | | **Stylo_80**  **VIC** | **Stylo_17**  **FAM-6** | **Stylo_48**  **PET** | | **Stylo_55**  **VIC** | **Stylo_82**  **NED** | |
| A | NA | 131/138 | 163/214 | | | 181/214 | 180/314 | 268/268 | | 292/297 | NA | |
|  |  |  |  |  |  |  |  |  |  |  |  |  |
| B | 256/275 | 131/131 | 175*/191 | | | 190/202 | 179/314 | 268/276 | | 278/278 | 227/243 | |
|  |  |  |  |  |  |  |  |  |  |  |  |  |
| C | NA | NA | NA | | | NA | 166/195 | NA | | 278/278 | 216/216 | |
|  |  |  |  |  |  |  |  |  |  |  |  |  |
| D | 267/267 | 110/115  103/134 |  | 163/206 | | 191/206 | 184/317 | 262/264 | | NA | 235/247 | |
|  |  |  |  | |  |  |  |  |  |  |  |  |
| E | 275/275 | 131/138 | 163/214 | | | 181/214 | 179/314 | 272/276 | | 214/278 | NA | |
|  |  |  |  |  |  |  |  |  |  |  |  |  |
| F | 263/263 | 111/115 | 175/210 | | | 181/190 | 172/186 | 264/264 | | NA | 235/247 | |
|  |  |  |  |  |  |  |  |  |  |  |  |  |
| G | 274/274 | 118/118 | 167/167 | | | 214/226 | 180/315 | 268/268 | | 292/298 | NA | |
|  |  |  |  |  |  |  |  |  |  |  |  |  |
| H | 277/277 | 107/115 | 206/214 | | | 182/286 | 185/202 | 273/278 | | 301/301 | 232/235 | |
|  |  |  |  |  |  |  |  |  |  |  |  |  |

Supplementary Table S2. Microsatellite allele sizes (base pair) in fragments (F) of *Stylophora pistillata* control (non-chimeric) colonies and their respective maternal colonies (M). Genotype I and II (GI, GII) show the base pair sizes of the 8 microsatellites used in four-channel capillary electrophoresis (Applied Biosystems® 3500). PET, FAM-6, NED and VIC are the fluorescent probes. NA = data not available due to no visible peaks or weak peaks (<100 fluorescence units) in the electropherogram.

| **Sample** | **GI** | | | | | | **GII** | | |  |
| --- | --- | --- | --- | --- | --- | --- | --- | --- | --- | --- |
|  | **Stylo_45**  **PET** | **Stylo_72**  **FAM-6** | **Stylo_73**  **NED** | **Stylo_80**  **VIC** | **Stylo_17**  **FAM-6** | **Stylo_48**  **PET** | | **Stylo_55**  **VIC** | **Stylo_82**  **NED** | |
| **M_A** | **NA** | **131/138** | **163/214** | **181/214** | **180/314** | **268/268** | | **292/297** | **NA** | |
| F_1 | NA | 119/138 | 163/214 | 181/194 | 180/180 | NA | | 278/297 | NA | |
| F_2 | NA | 119/138 | 163/214 | 181/194 | 180/180 | NA | | 278/297 | NA | |
| F_3 | NA | 119/138 | 163/214 | 181/194 | 180/180 | NA | | 278/297 | NA | |
| F_4 | NA | 119/138 | 163/214 | 181/194 | 180/180 | NA | | 278/297 | NA | |
| F_5 | NA | 119/138 | 163/214 | 181/194 | 180/180 | NA | | 278/297 | NA | |
| F_6 | NA | 119/138 | 163/214 | 181/194 | 180/180 | NA | | 278/297 | NA | |
| F_7 | NA | 119/138 | 163/214 | 181/194 | 180/180 | NA | | 278/297 | NA | |
| Ft_8 | NA | 119/138 | 163/214 | 181/194 | 180/180 | NA | | 278/297 | NA | |
| **M_E** | **275/275** | **131/138** | **163/214** | **181/214** | **179/314** | **272/276** | | **214/278** | **NA** | |
| F_1 | 275/275 | 131/146 | 187/214 | 202/214 | 179/314 | 268/276 | | 278/278 | NA | |
| F_2 | 275/275 | 131/146 | 187/214 | 202/214 | 179/314 | 268/276 | | 278/278 | 216/216 | |
| F_3 | 275/275 | 131/146 | 187/214 | 202/214 | 179/314 | 268/276 | | 278/278 | 216/216 | |
| F_4 | 275/275 | 131/146 | 187/214 | 202/214 | 179/314 | 268/276 | | 278/278 | 216/216 | |
| F_5 | 275/275 | 131/146 | 187/214 | 202/214 | 179/314 | 268/276 | | 278/278 | 216/216 | |
| F_6 | 275/275 | 131/146 | NA | 202/214 | 179/314 | NA | | 278/278 | NA | |

Supplementary Table S3. Microsatellite allele sizes (base pairs) in fragments (F) of chimeric *Stylophora pistillata* colonies that were not elucidated as chimeras by microsatellites and their respective maternal colonies (M). Genotype I and II (GI, GII) show the bp sizes of the 8 microsatellites used in four-channel capillary electrophoresis (Applied Biosystems® 3500). PET, FAM-6, NED and VIC are the fluorescent probes. NA = data not available due to no visible peaks or weak peaks (<100 fluorescence units) in the electropherogram.

| **Chimera** | **Sample** | **GI** | | | | | **GII** | | | |  |
| --- | --- | --- | --- | --- | --- | --- | --- | --- | --- | --- | --- |
|  |  | **Stylo_45**  **PET** | **Stylo_72**  **FAM-6** | **Stylo_73**  **NED** | **Stylo_80**  **VIC** | **Stylo_17**  **FAM-6** | | **Stylo_48**  **PET** | **Stylo_55**  **VIC** | **Stylo_82**  **NED** | |
| **03** | **M_D** | **267/267** | **110/115** | **163/206** | **191/206** | **184/317** | | **262/264** | **NA** | **235/247** | |
|  |  |  |  |  |  |  |  |  |  |  |  |
|  | F_1 | 267/267 | 110/115 | 163/206 | 191/198 | 184/184 | | 264/264 | NA | 231/247 | |
|  | F_2 | 267/267 | 110/115 | 163/202 | 191/198 | 184/184 | | 264/264 | NA | 231/247 | |
|  | F_3 | 267/267 | 110/115 | 163/202 | 191/198 | 184/184 | | 264/264 | NA | 231/247 | |
|  | F_4 | 267/267 | 110/115 | 163/202 | 191/198 | 184/184 | | 264/264 | NA | 231/247 | |
|  | F_5 | 267/267 | 110/115 | 163/202 | 191/198 | 184/184 | | 264/264 | NA | 231/247 | |
|  | F_6 | 267/267 | 110/115 | 163/202 | 191/198 | 184/184 | | 264/264 | NA | 231/247 | |
|  | F_7 | 267/267 | 110/115 | 163/202 | 191/198 | 184/184 | | 264/264 | NA | 231/247 | |
|  | F_8 | 267/267 | 110/115 | 163/202 | 191/198 | 184/184 | | 264/264 | NA | 231/247 | |
|  | F_9 | 267/267 | 110/115 | 163/202 | 191/198 | 184/184 | | 264/264 | NA | 231/247 | |
|  | F_10 | 267/267 | 110/115 | 163/202 | 191/198 | 184/184 | | 264/264 | NA | 231/247 | |
|  | F_11 | 267/267 | 110/115 | 163/202 | 191/198 | 184/184 | | 264/264 | NA | 231/247 | |
|  | F_12 | 267/267 | 110/115 | 163/202 | 191/198 | 184/184 | | 264/264 | NA | 231/247 | |
|  | F_13 | 267/267 | 110/115 | 163/202 | 191/198 | 184/184 | | 264/264 | NA | 231/247 | |
|  | F_14 | 267/267 | 110/115 | 163/202 | 191/198 | 184/184 | | 264/264 | NA | 231/247 | |
|  | F_15 | 267/267 | 110/115 | 163/202 | 191/198 | 184/184 | | 264/264 | NA | 231/247 | |
|  | F_16 | 267/267 | 110/115 | 163/202 | 191/198 | 184/184 | | 264/264 | NA | 231/247 | |
|  | F_17 | 267/267 | 110/115 | 163/202 | 191/198 | 184/184 | | 264/264 | NA | 231/247 | |
| **44** | **M_C** | **NA** | **NA** | **NA** | **NA** | **166/195** | | **NA** | **278/278** | **216/216** | |
|  |  |  |  |  |  |  |  |  |  |  |  |
|  | F_1 | 277/277 | 131/174 | 171/179 | 186/190 | 196/314 | | NA | 278/278 | 216/228 | |
|  | F_2 | 277/277 | 131/174 | 171/179 | 186/190 | 196/314 | | NA | 278/278 | 216/228 | |
|  | F_3 | 277/277 | 131/174 | 171/179 | 186/190 | 196/314 | | NA | 278/278 | 216/228 | |
|  | F_4 | 277/277 | 131/174 | 171/179 | 186/190 | 196/314 | | NA | 278/278 | 216/228 | |
|  | F_5 | 277/277 | 131/174 | 171/179 | 186/190 | 196/314 | | NA | 278/278 | 216/228 | |
|  | F_6 | 277/277 | 131/174 | 171/179 | 186/190 | 196/314 | | NA | 278/278 | 216/228 | |
|  | F_7 | 277/277 | 131/174 | 171/179 | 186/190 | 196/314 | | NA | 278/278 | 216/228 | |
|  | F_8 | 277/277 | 131/174 | 171/179 | 186/190 | 196/314 | | NA | 278/278 | 216/228 | |
|  | F_9 | 277/277 | 131/174 | 171/179 | 186/190 | 196/314 | | NA | 278/278 | 216/228 | |
|  | F_10 | 277/277 | 131/174 | 171/179 | 186/190 | 196/314 | | NA | 278/278 | 216/228 | |
|  | F_11 | 277/277 | 131/174 | 171/179 | 186/190 | 196/314 | | NA | 278/278 | 216/228 | |
|  | F_12 | 277/277 | 131/174 | 171/179 | 186/190 | 196/314 | | NA | 278/278 | 216/228 | |
|  | F_13 | 277/277 | 131/174 | 171/179 | 186/190 | 196/314 | | NA | 278/278 | 216/228 | |
|  | F_14 | 277/277 | 131/174 | 171/179 | 186/190 | 196/314 | | NA | 278/278 | 216/228 | |
| **49** | **M_B** | **256/275** | **131/131** | **175*/191** | **190/202** | **179/314** | | **268/276** | **278/278** | **227/243** | |
|  |  |  |  |  |  |  |  |  |  |  |  |
|  | **M_F** | **263/263** | **111/115** | **175/210** | **181/190** | **172/186** | | **264/264** | **NA** | **235/247** | |
|  |  |  |  |  |  |  |  |  |  |  |  |
|  | F_1 | 275/275 | 131/178 | 167/179* | 190/194 | 179/314 | | NA | 278/278 | 216/243 | |
|  | F_2 | 275/275 | 131/178 | 167/179* | 190/194 | 179/314 | | NA | 278/278 | 216/243 | |
|  | F_3 | 275/275 | 131/178 | 167/179* | 190/194 | 179/314 | | NA | 278/278 | 216/243 | |
|  | F_4 | 275/275 | 131/178 | 167/179* | 190/194 | 179/314 | | NA | 278/278 | 216/243 | |
|  | F_5 | 275/275 | 131/178 | 167/179* | 190/194 | 179/314 | | NA | 278/278 | 216/243 | |
|  | F_6 | 275/275 | 131/178 | 167/179* | 190/194 | 179/314 | | NA | 278/278 | 216/243 | |
|  | F_7 | 275/275 | 131/178 | 167/179* | 190/194 | 179/314 | | 268/276 | 278/278 | 216/243 | |
|  | F_8 | 275/275 | 131/178 | 167/179* | 190/194 | 179/314 | | 268/276 | 278/278 | 216/243 | |
|  | F_9 | 275/275 | 131/178 | 167/179* | 190/194 | 179/314 | | 268/276 | 278/278 | 216/243 | |
|  | F_10 | 275/275 | 131/178 | 167/179* | 190/194 | 179/314 | | 268/276 | 278/278 | 216/243 | |
|  | F_11 | 275/275 | 131/178 | 167/179* | 190/194 | 179/314 | | 268/276 | 278/278 | 216/243 | |
|  | F_12 | 275/275 | 131/178 | 167/179* | 190/194 | 179/314 | | 268/276 | 278/278 | 216/243 | |
|  | F_13 | 275/275 | 131/178 | 167/179* | 190/194 | 179/314 | | 268/276 | 278/278 | 216/243 | |
|  | F_14 | 275/275 | 131/178 | 167/179* | 190/194 | 179/314 | | 268/276 | 278/278 | 216/243 | |
|  | F_15 | 275/275 | 131/178 | 167/179* | 190/194 | 179/314 | | 268/276 | 278/278 | 216/243 | |
|  | F_16 | 275/275 | 131/178 | 167/179* | 190/194 | 179/314 | | 268/276 | 278/278 | 216/243 | |
|  | F_17 | 275/275 | 131/178 | 167/179* | 190/194 | 179/314 | | 268/276 | 278/278 | 216/243 | |
| **74** | **M_E** | **275/275** | **131/138** | **163/214** | **181/214** | **179/314** | | **272/276** | **214/278** | **NA** | |
|  |  |  |  |  |  |  |  |  |  |  |  |
|  | F_1 | 275/275 | 107/131 | 163/179 | 181/181 | 176/314 | | 268/276 | 278/292 | 216/243 | |
|  | F_2 | 275/275 | 107/131 | 163/179 | 181/181 | 176/314 | | 268/276 | 278/292 | 216/243 | |
|  | F_3 | 275/275 | 107/131 | 163/179 | 181/181 | 176/314 | | 268/276 | 278/292 | 216/243 | |
|  | F_4 | 275/275 | 107/131 | 163/179 | 181/181 | 176/314 | | 268/276 | 278/292 | 216/243 | |
|  | F_5 | 275/275 | 107/131 | 163/179 | 181/181 | 176/314 | | 268/276 | 278/292 | 216/243 | |
|  | F_6 | 275/275 | 107/131 | 163/179 | 181/181 | 176/314 | | 268/276 | 278/292 | 216/243 | |
|  | F_7 | 275/275 | 107/131 | 163/179 | 181/181 | 176/314 | | 268/276 | 278/292 | 216/243 | |
|  | F_8 | 275/275 | 107/131 | 163/179 | 181/181 | 176/314 | | 268/276 | 278/292 | 216/243 | |
|  | F_9 | 275/275 | 107/131 | 163/179 | 181/181 | 176/314 | | 268/276 | 278/292 | 216/243 | |
|  | F_10 | 275/275 | 107/131 | 163/179 | 181/181 | 176/314 | | 268/276 | 278/292 | 216/243 | |
|  | F_11 | 275/275 | 107/131 | 163/179 | 181/181 | 176/314 | | 268/276 | 278/292 | 216/243 | |
|  | F_12 | 275/275 | 107/131 | 163/179 | 181/181 | 176/314 | | 268/276 | 278/292 | 216/243 | |
|  | F_13 | 275/275 | 107/131 | 163/179 | 181/181 | 176/314 | | 268/276 | 278/292 | 216/243 | |
|  | F_14 | 275/275 | 107/131 | 163/179 | 181/181 | 176/314 | | 268/276 | 278/292 | 216/243 | |
|  | F_15 | 275/275 | 107/131 | 163/179 | 181/181 | 176/314 | | 268/276 | 278/292 | 216/243 | |
|  | F_16 | 275/275 | 107/131 | 163/179 | 181/181 | 176/314 | | 268/276 | 278/292 | 216/243 | |
|  | F_17 | 275/275 | 107/131 | 163/179 | 181/181 | 176/314 | | 268/276 | 278/292 | 216/243 | |
|  | F_18 | 275/275 | 107/131 | 163/179 | 181/181 | 176/314 | | 268/276 | 278/292 | 216/243 | |
|  | F_19 | 275/275 | 107/131 | 163/179 | 181/181 | 176/314 | | 268/276 | 278/292 | 216/243 | |
|  | F_20 | 275/275 | 107/131 | 163/179 | 181/181 | 176/314 | | 268/276 | 278/292 | 216/243 | |
| **101** | **M_H** | **277/277** | **107/115** | **206/214** | **182/286** | **185/202** | | **273/278** | **301/301** | **232/235** | |
|  |  |  |  |  |  |  |  |  |  |  |  |
|  | F_1 | 277/277 | 107/115 | 206/214 | 182/198 | 190/202 | | 264/273 | NA | 232/232 | |
|  | F_2 | 277/277 | 107/115 | 206/214 | 182/198 | 190/202 | | 264/273 | NA | 232/232 | |
|  | F_3 | 277/277 | 107/115 | 206/214 | 182/198 | 190/202 | | 264/273 | NA | 232/232 | |
|  | F_4 | 277/277 | 107/115 | 206/214 | 182/198 | 190/202 | | 264/273 | NA | 232/232 | |
|  | F_5 | 277/277 | 107/115 | 206/214 | 182/198 | 190/202 | | 264/273 | NA | 232/232 | |
|  | F_6 | 277/277 | 107/115 | 206/214 | 182/198 | 190/202 | | 264/273 | NA | 232/232 | |
|  | F_7 | 277/277 | 107/115 | 206/214 | 182/198 | 190/202 | | 264/273 | NA | 232/232 | |
|  | F_8 | 277/277 | 107/115 | 206/214 | 182/198 | 190/202 | | 264/273 | NA | 232/232 | |
|  | F_9 | 277/277 | 107/115 | 206/214 | 182/198 | 190/202 | | 264/273 | NA | 232/232 | |
|  | F_10 | 277/277 | 107/115 | 206/214 | 182/198 | 190/202 | | 264/273 | NA | 232/232 | |
|  | F_11 | 277/277 | 107/115 | 206/214 | 182/198 | 190/202 | | 264/273 | NA | 232/232 | |
